# Bioaccumulation and biomagnifications of toxic metals in tissues of loggerhead turtles (*Caretta caretta*) from the Mediterranean Sea coast, Egypt

**DOI:** 10.1038/s41598-023-33972-9

**Published:** 2023-05-17

**Authors:** Maha Ahmed Mohamed Abdallah

**Affiliations:** National Institute of Oceanography and Fisheries, NIOF, Cairo, Egypt

**Keywords:** Chemical biology, Ecology, Environmental sciences

## Abstract

Heavy metal concentrations in the different tissues of marine turtles are presented; the most frequently monitored elements are mercury, cadmium, and lead. Concentrations of Hg, Cd, Pb, and As in different organs and tissues (liver, kidney, muscle tissue, fat tissue, and blood) of loggerhead turtle *Caretta caretta* from the southeastern Mediterranean Sea were determined using Atomic Absorption Spectrophotometer, Shimadzu and mercury vapor unite (MVu 1A) for Hg measurements. The highest levels of cadmium and arsenic were found in the kidney (Cd: 61.17 µg g^−1^; As: 0.051 µg g^−1^ dry weight). For lead, the highest level was found in muscle tissue (35.80 µg g^−1^). Mercury tended to be higher in the liver than in other tissues and organs (0.253 µg g^−1^ dry weight) which showed a higher accumulation of this element. Fat tissue generally displays the lowest trace element burdens. The concentrations of As remained low in all the considered tissues, possibly the result of low trophic levels in sea turtles. In contrast, the diet of loggerhead turtles would result in significant exposure to Pb. This is the first study into metal accumulation in the tissues of a loggerhead turtle from the Egyptian Mediterranean coastline.

## Introduction

Several studies have been carried out concerning metal accumulation in sea turtles from different areas of the world; studies in the southeastern Mediterranean Sea are limited. The loggerhead sea turtle is suggested to be considered ‘‘endangered’’ in the Mediterranean Sea. A world-wide, reduction of marine turtle populations has been attributed to different factors such as coastal pollution, due to the production of waste and pollutants generated by different human activities (industrial and domestic), which makes places unsuitable for nesting^1^, accidental capture and the use of eggs and meat as food. Recent reports showed, that sea turtles are affected by marine pollutants, such as debris, tar balls^2,3^ and toxic chemicals, such as heavy metals^4,5^. Among the wide range of toxic chemicals, trace metals including both essential and non-essential elements, have a particular significance in ecotoxicology. According to their chemical characteristics (e.g., lipophilia), heavy metals tend to bioaccumulate and biodegrade differently in tissues^6^ and, unlike organic pollutants such as pesticides, are not biodegradable, since heavy metals are highly persistent and all have the potential to be toxic to living organisms. Moreover, it has been observed that the increase in such contamination could adversely interfere with fertilization and the success of hatching^7–10^.

The knowledge of trace metal burden in marine turtles is, therefore, an important focal point to assess the potential impact of these compounds on these endangered organisms. Therefore, the study of toxic metals in C. *caretta* is fundamental to evaluating their potentially toxic effects and estimating the type and the degree of exposure. In the same context, systemic toxic effects, such as teratogenicity, cytotoxicity, and genotoxicity, can cause multiple damages to the reproductive, immune, and nervous systems, and behaviors, and may cause carcinogenesis. However, the database in terms of such contaminants available for the Mediterranean turtle population is very limited^4,11^; though the importance of monitoring trace metal burden in marine turtles in an effort to conserve their population is clear^12^. Because of their solitary lifestyle, duration of the pelagic phase and, long-lasting apneas, sea turtles are in fact among the most difficult marine animals to assess^13^. Two of the seven species of sea turtles are known to reproduce regularly in the Mediterranean Sea, the loggerhead turtle, *Caretta caretta*, and the green turtle, Chelonia mydas^14^.

Turtles feed primarily on sessile or slowly moving benthic prey^15^ although they do capture organisms throughout the water column^16^. The loggerhead turtle is currently classified as ‘‘endangered’’ by the UCN (International Union for the Conservation of Nature and Natural Resources).

Small populations of *Caretta caretta* and C. mydas nesting on the northern Sinai Peninsula are currently under intense pressure from human activities. The fact that 10 nests exhibited signs of human pilfering of eggs is particularly disturbing considering that only 21 egg depositions could be confirmed for the entire season. The combined effects of the capture of adults at sea (caught by trawling and bottom longline fishing), predation of eggs, coastal pollution and development of beaches, threaten to exclude nesting turtles from the Mediterranean coastline of Egypt within the next decade. An intense public awareness campaign and a conservation program of nest protection and/or re-location are required immediately if this population is to survive^1^.

The primary objective of this study was assess specific metal accumulation in various tissues and organs of loggerhead turtles from the southeastern Mediterranean Sea measuring the concentrations of the potentially toxic elements, Cd, Pb, Hg and As, and to compare our data with those reported from other locations, and improve the knowledge of these contaminants and their potential impact on this important species. Correlations between the elements were determined in order to investigate physiological disorders and the hazard that these contaminants may pose to loggerhead turtles’ survival was discussed. On the other hand, by using the concentrations of trace metals in the tissues of these animals, we can learn about their locations in the ocean and their transmission.

## Materials and methods

### Sample collection

**Figure 1:**
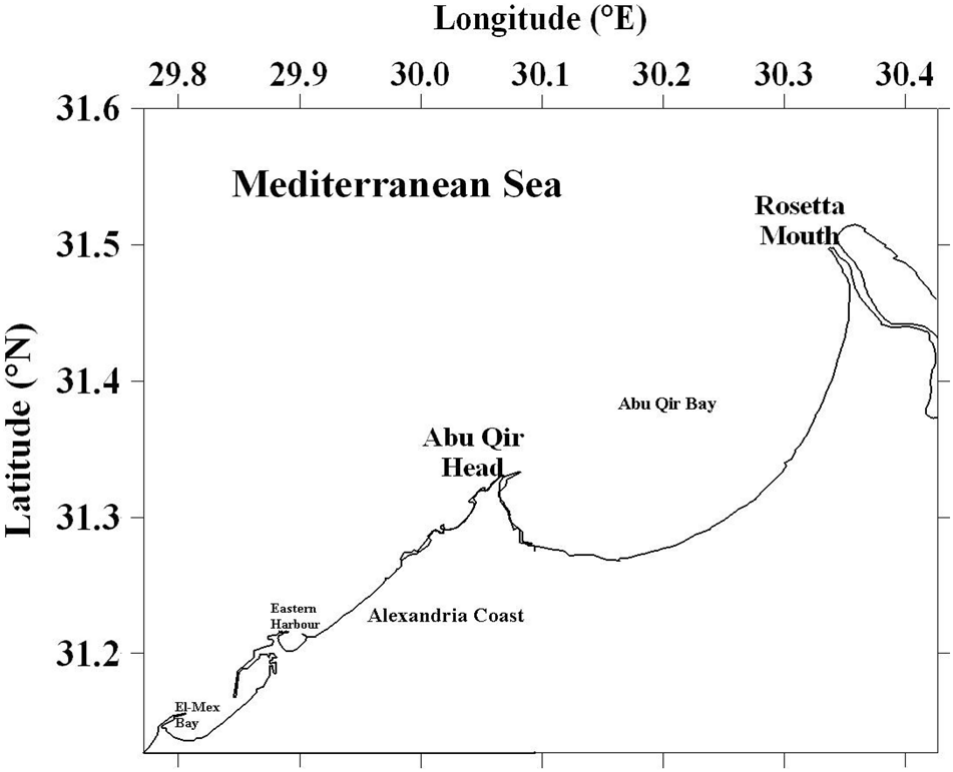
Sampling sites from Alexandria coast of Egyptian Mediterranean Sea.

Loggerhead turtles (n = 8) from Alexandria coast, Egypt in June 2019 and August 2019 were caught by fishermen without permission for commercial use near Abu Qir Bay (Fig. 1). Standard length and width were measured. The means of length, width were 75.6 cm (range: 70.6–85.8 cm) and 51.6 cm (46.2–55.3 cm) respectively. Kidney, liver, muscle, fat and blood were collected and were kept at − 4 °C until analysis. Blood samples were taken from the live animals.

### Preparation of samples for metal analyses

To avoid contamination, all reagents were handled carefully, polyethylene disposables were thoroughly washed with HNO_3_ 2 N under a fume hood and disposable gloves were worn during the procedure. All of the reagents used were Suprapur (Merck) and the water was bidistilled and deionized (Mili-Q). Different tissues was digested using a high-temperature digestion with a mixture of nitric, perchloric, and sulphuric acids (8:8:1), following the method described by García-Fernández et al.^17^. The Teflon tubes used (100 mm) for wet digestion were previously washed with 2% nitric acid for 48 h and then rinsed twice with Deionized double distilled water (DDDW) and dried in an oven at 50 °C. Approximately 1.5 g of wet tissue of each sample was dried in an oven at 80 °C until a constant dry weight was obtained. A weight of 0.5 g of these dry tissues was placed in the Teflon digestion tube, to which 5 ml of the acid mixture was added. Samples were digested with a thermostatic apparatus (microwave) equipped for controlling temperatures from 10 to 250 °C and an aluminum-heating block. Thermal digestion was performed by raising the temperature from 100 to 200 °C at a rate of 5 °C/min, followed by digestion at 200 °C for 2 h, and a further temperature increase to 250 °C at a rate of 20 °C/5 min; this temperature was maintained until complete dryness was obtained.

After collection, all blood samples were stored at 4 °C and were analyzed within 2 days. Two hours before sample preparation, the blood samples were brought to room temperature. They were mixed gently for homogenization and 500 µL of the blood sample were diluted with 100µL 0.1% (V/V) Triton-X-100 solution and 500 µL of the internal standard solution. This solution was filled up to 5 mL with a 0.5% (V/V) NH_4_OH solution in a 10 mL autosampler polypropylene tube using a 5 mL bottle-top dispenser. Finally, 5 mL of conc. HNO_3_ was added at each sample in a 50-mL closed PTFE tube and heated at 120 ± 1 °C for 1 h for the decomposition of the organic structure. The closed tubes were cooled and opened when the internal temperature of the high-pressure bomb was under 30 °C. The digests were quantitatively transferred to 25-mL volumetric flasks and diluted to volume with de-ionized water.

### Analysis of metals

The digested sample was allowed to cool; finally, Deionized double distilled water was added to the resulting samples to bring their volume to 25 mL and they were analyzed with AAS (AA-6800 Atomic Absorption Spectrophotometer, Shimadzu) for Cd, Pb, As and Hg. Concentrations of (As) were measured using Hydride Vapor Generation for As and concentrations of (Hg) were measured by mercury vapor unite (MVu 1A—Mercury vaporizer unite) only used for Hg. Results were expressed as microgram of element per dry weight gram of different tissues, detection limit was estimated in ≤ 0.05 ppp wet weight for Hg, 0.01, 0.02 and 0.02 ppm wet weight for Cd, Pb and As respectively.

Accuracy of this analysis was examined using standard reference materials SRM Dorm-2 (National Research Council Canada). Recoveries of all the elements ranged from 88 to 109%.

### Statistical analysis

One way analysis of variance (ANOVA) and Duncan's test (p = 0.05) were used to access whether trace elements concentrations varied significantly. Spearman correlation between heavy metals within the tissues of the loggerheads turtles was determined. All statistical calculations were performed with SPSS 9.0 for windows. A p value of less than 0.05 was considered to indicate statistical significance.

### Ethics approval

This is an observational study. The Research Ethics Committee has confirmed that no ethical approval is required.”

### Consent to participate

Informed consent was obtained from all individual participants included in the study. (it is single author Maha Ahmed Mohamed Abdallah only).

## Results and discussion

### Metals in sea turtle tissues and comparison with literature data

Concentrations of Cd, Pb, As and Hg (µg/g dry weight) in muscle, liver kidney, fat and blood of loggerhead turtles are presented in Table 1.

**Table 1:** Element concentrations range and (average ± SD) in various tissues and organs of loggerhead turtles from the Egyptian Mediterranean coast (µg/g, dry wt.).

Metal	n	Muscle	Liver	Kidney	Fat	Blood (µg/ml)
Cd	8	0.50–2.0	1.57–10.95	30.96–61.17	0.138–0.391	6.21–6.67
		(0.97 ± 0.54)	(6.45 ± 3.95)	(42.49 ± 11.61)	(0.265 ± 0.09)	(6.645 ± 2.55)
Pb	8	4.70–35.80	3.29–16.32	3.52–9.71	1.36–2.55	1.85–3.19
		(11.23 ± 2.10)	(6.12 ± 1.22)	(6.51 ± 2.12)	(2.01 ± 0.43)	(2.46 ± 0.49)
As	8	0.21–0.33	0.1–1.112	0.08–0.51	0.07–0.106	1.56–6.42
		(0.267 ± 0.04)	(0.94 ± 0.01)	(0.272 ± 0.014)	(0.098 ± 0.02)	(3.435 ± 1.71)
Hg	8	0.32–0.61	0.39–2.53	0.39–1.29	0.02–0.05	1.44–4.75
		(0.47 ± 0.09)	(1.16 ± 0.76)	(0.88 ± 0.34)	(0.04 ± 0.01)	(3.347 ± 0.67)

### Cadmium

Cadmium concentrations in muscles of loggerhead turtles were slightly higher (0.47 ± 0.09 µg/g) than most of the levels in specimens from the Mediterranean Sea^17–20^, Table 2, but lower than that from Italian Adriatic coasts^21,22^ and from North Tunisian Coasts recorded by Sami et al.^23^. A number of hypotheses might explain the variability in Cd load among loggerhead turtles from the different areas. Firstly, the environmental contamination that influences in the food chain of these organisms and secondly, the age of the specimens sampled. Cadmium is known to accumulate in marine vertebrate with age. The age is, in fact, known to be one of factors which control concentration of Cd in marine vertebrates^24,25^ i.e. the change in diet during the different stages of animal growth.

**Table 2:** Concentrations of toxic metals ( µg/g dry wt) in different tissues of Loggerhead Turtles (*Caretta* *caretta*) from various locations.

Location	Organ	Cd		Pb		As		Hg	
		n	Mean ± SD	N	Mean ± SD	n	Mean ± SD	n	Mean ± SD
West Italy^a^	Muscle	26	0.20 ± 0.2		–		–	26	0.40 ± 0.30
Adriatic Sea^b^		17	1.28 ± 0.11		–		–	–	
East Mediterranean^c^		19	0.25 ± 0.03	19	0.14 ± 0.03		–	19	0.64 ± 0.021
West Mediterranean^d^		6	0.11 ± 0.07	6	0.25 ± 0.27	6	37.73 ± 31.44	6	0.24 ± 0.30
Southwestern Mediterranean^e^		20	0.20 ± 0.14	20	0.26 ± 0.23		–	–	
Adriatic and Mediterranean^f^		10	0.81 ± 0.04		BLD		–	–	
North Tunisian Coasts^i^		4	0.80 ± 1.13	4	0.26 ± 0.30		–	4	0.12 ± 0.13
Present study		8	0.47 ± 0.09	8	11.23 ± 2.10	8	0.269 ± 0.04	8	0.47 ± 0.09
West Italy^a^	Liver	14	19.3 ± 34.2	–		–		22	1.10 ± 1.70
East Mediterranean^c^		19	7.79 ± 1.94	19	0.37 ± 0.05	–		19	0.99 ± 0.29
West Mediterranean^d^		3	0.63 ± 0.5	3	0.17 ± 0.13	3	16.39 ± 19.02	3	0.14 ± 0.04
Southwestern Mediterranean^e^		16	23.3 ± 53.6	16	2.75 ± 1.64	–		–	
Adriatic and Mediterranean^f^		11	2.4 ± 0.4	11	0.1 ± 0.08	–		–	
Sicilian coast-Mediterranean^h^		8	10.2 ± 11.0	8	0.12 ± 0.24	8	77.27 ± 45.21	8	1.05 ± 0.89
North Tunisian Coasts^i^		5	8.31 ± 3.22	5	0.23 ± 0.01	–		5	1.15 ± 0.8
Present study		8	6.45 ± 3.95	8	6.12 ± 1.22	8	0.94 ± 0.01	8	1.16 ± 0.76
West Italy^a^	Kidney	19	57.2 ± 34.6	–		–		20	0.90 ± 0.70
East Mediterranean^c^		19	26.50 ± 4.83	19	0.38 ± 0.07	–		19	0.51 ± 0.007
West Mediterranean^d^		4	8.24 ± 7.01	4	1.27 ± 1.12	4	34.24 ± 42.86	4	0.45 ± 0.59
Southwestern Mediterranean^e^		19	31.47 ± 70.75	19	0.52 ± 0.49	–		–	
Adriatic and Mediterranean^f^		9	5.80 ± 1.10	9 0.1 ± 0.07		–		–	
Sicilian coast-Mediterranean^h^		3	39.90 ± 41.86	3	0.19 ± 0.32	3	166.17 ± 74.74	3	0.44 ± 0.36
North Tunisian Coasts^i^		2	78.13 ± 59.6	3	0.67 ± 0.51	–		3	0.61 ± 0.39
Present study		8	42.49 ± 11.61	8	6.51 ± 2.12	8	0.272 ± 0.014	8	0.88 ± 0.34
East Mediterranean^c^	Fat	19	0.10 ± 0.06	19	0.11 ± 0.03	–		19	0.05 ± 0.03
Sicilian coast-Mediterranean^h^		3	0.12 ± 0.13	3	< 0.02	3	32.15 ± 27.0	3	0.04 ± 0.01
Present study		8	0.39 ± 0.09	8	2.01 ± 0.43	8	0.098 ± 0.02	8	0.04 ± 0.01
West Mediterranean^d^	Blood	5	0.12 ± 0.21	5	0.31 ± 0.31	5	6.99 ± 9.28	5	0.02 ± 0.01
USA^g^			–		–	–			0.029 ± 0.008
Present study		8	6.645 ± 2.55	8	2.46 ± 0.49	8	3.735 ± 1.710	8	3.347 ± 0.670

Values reported in wet weight were converted to dry weight using the mean water content as determined in present study. BLD: Below the detection limit.

^a^Maffucci et al.^18^; ^b^Franzellitti et al.^21^; ^c^Storelli et al.^19^; ^d^Jerez et al.^20^; ^e^García-Fernández et al.^17^; ^f^Andreani et al.^22^.

^g^Day et al.^40^; ^h^Dario et al.^10^; ^i^Sami et al.^23^.

**Figure 2:**
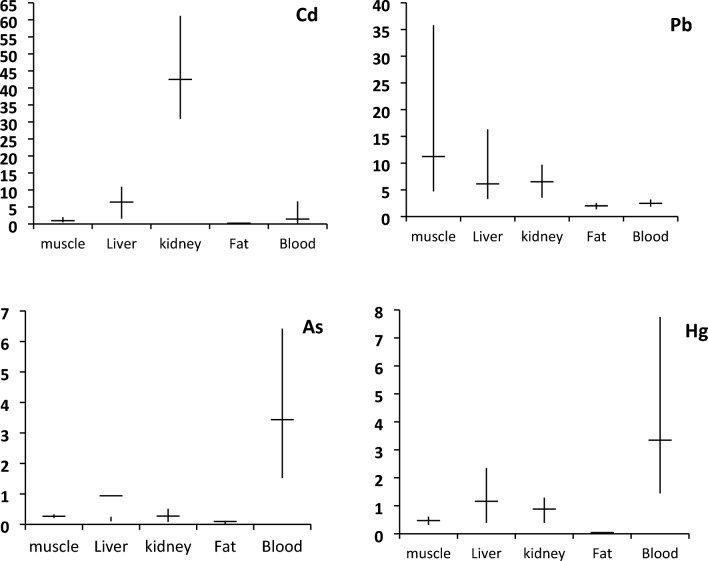
Concentration of heavy metals in different tissues of *Caretta caretta* from the the Egyptian Mediterranean coast.

Cd concentrations in liver (6.45 ± 3.95 µg/g) were similar to those detected in blood (6.645 ± 2.55 µg/g) (p > 0.05, Fig. 2), (Table 2) showed that the specimens here analyzed had hepatic levels of Cd lower than those reported for loggerhead turtles in other parts of the Mediterranean Sea^10,17,18,23^, except for animals from eastern Mediterranean Sea^19^, which showed comparable levels while animals from western Mediterranean^20,22^ showed low levels. Thus, the high hepatic of Cd in the organisms in question seems to indicate a recent exposure to a contamination source.

On the other hand, the highest mean concentration of Cd was measured in kidney of loggerhead turtles (Cd_kidney_/Cd_liver_ = 6.59). This provides evidence of a tendency for large scale accumulation of Cd in kidney tissue. The continuous sorption of small quantities of Cd, which is typical of chronic exposure, gives rise to a prevalence of levels in the kidney over those in the liver^20^, on account of the large period of time that this metal stays in the kidney due to poor elimination. A comparison with literature data (Table 2) showed that the specimens here analyzed had renal levels of Cd higher than those reported for loggerhead turtles in other parts of the Mediterranean Sea. Meanwhile, the level of Cd to some extent is similar to the levels in specimens from the western and southwestern Mediterranean Sea^17,18^, and lower than that recorded in West Italy and North Tunisian Coasts by^18,23^. However, independently from the many factors influencing Cd accumulation in marine turtles, the observed pattern of metal distribution between liver and kidney does not match well with what generally encountered in marine turtles, where kidney exhibits the highest Cd concentrations^18,19,26^. Studies on fresh water turtles by Rie et al.^27^ reveal that, liver is the primary site of Cd accumulation upon short-term exposure, whereas during long-term exposure, Cd is redistributed from liver, via the blood, to kidney where it is absorbed and concentrated, this may explain the high level of Cd in the blood in the present study. Accordingly, our results strongly agreed with^27^ in particular because our search was on the adult animals. Furthermore, the continuous sorption of small quantities of Cd which is typical of chronic exposure, gives rise to a prevalence of levels in the kidney over those in the liver, on account of the large period of time that this metal stays in the kidney due to poor elimination. Low accumulation of Cd (0.391 ± 0.09 µg/g) was observed in fat (Fig. 2). Few data are available as to the levels of this metal in turtle’s fat. As reported in in this study, cadmium tends to bioaccumulated more in kidneys and liver than in muscles and in fat. In addition, it is estimated that, in case of chronic exposure, the renal concentration of cadmium (poorly eliminated) would be higher than that of the liver. Concerning toxic effects, a cadmium chloride exposure in kidneys were high enough to adversely affect the health of sea turtles such as *C. caretta*.

It is difficult to put forward any detailed hypothesis which could explain these differences between contamination levels in loggerhead turtles from different locations. Marine environment quality differences between the locations are one possibility, leading to an unequal intake of Cd via the trophic route. Alternatively we should also consider the variation in the capacity to accumulate Cd which may be related to age.

### Lead

In the present study, we found that the concentration of lead in the muscles of *Caretta caretta* is approximately two times higher than that in the liver and kidneys, and about 5 times higher than that in fat and blood. The mean for Pb was five and 11.5 times, respectively, higher in fat and muscle than that of Cd (Table 1, Fig. 2). Generally, Pb exhibited high mean concentration in all studied tissues. Storelli et al.^19^ were of the opinion that lead concentrations below 0.5 µg/g (w.w.) should be considered low. In the present study, 100% of all studied tissues analyzed (n = 8) showed Pb concentrations much higher than this level. Comparison of lead concentrations between our study and previous data regarding loggerhead turtles sampled in the Adriatic Sea and different areas of the Mediterranean Sea (Table 2) reveals a higher contamination degree in animals analyzed here. Lead is a multi-targeted toxicant, and inorganic lead is carcinogenic in animals^28,29^. In all loggerhead turtle tissues reported from different authors, in Table 2, Pb levels were of the same order of magnitude, except those reported by^17,20^ for Liver and kidney respectively. Lam et al.^30^ reveal that, the muscle of adults from South China had relatively higher Pb concentrations than the other samples.

According to these authors, this drop was mainly due to the reduction of leaded petrol in many European countries since the 1970s^19^. Confirmation of^31^ that the low concentrations of Pb in animals tissues confirms the decrease in Pb pollution in the Mediterranean, due to the limitation of its use as an additive in gasoline. In the present study, lead concentrations in all tissues were much higher than those described by Storelli et al. ^19^; however, given that we have no data prior to the abolishment of leaded petrol in Egypt, we cannot know if the same tendency towards a decrease has also taken place.

### Mercury

Mercury concentrations in all tissues and organs of the specimens analyzed were, to some extent, similar to the levels in loggerhead turtles from Mediterranean Sea. Sea turtles, in comparison with marine mammals^32,33^ , which, similar to marine reptiles, are at the top of food chains, contained low mercury concentrations, particularly in fat. The low body burden of mercury in these high trophic level organisms could be mainly attributed to the nature of their diet.

However, the lowest mercury levels were found in the fat tissue (0.04 ± 0.01 µg/g, dry wt**)** in agreement with what was seen by Sakai et al.^34^. This finding is surprising if one considers the lipophilic nature of methyl mercury, organic form of mercury primarily accumulated by marine organisms. Nevertheless, a suitable explanation for low levels of mercury in fat tissue may be provided by the large chemical affinity of methyl mercury for the—SH group of some proteins that, resulting in the loss of lipophilicity of the compound, makes the distribution of methyl mercury in tissues not more affected by their lipid content^35^. This ensures with the present study, where the relatively high concentrations of Hg was found in the tissues of liver (1.16 ± 0.76), kidney (0.88 ± 0.34) and muscle (0.47 ± 0.09) (Fig. 2).

The stability of Hg in the different tissues (as muscles, kidney, fat and liver) makes this tissue preferable for approximating long-term exposure, while blood Hg levels can be affected by recent changes in Hg intake. A large range in blood Hg concentrations (1.44–4.75 µg/ml) was found among species (Table 1), with concentrations rising for all species. This suggests that there is an elevation of bioavailable Hg in near shore habitats where terrestrial influences and anthropogenic impacts are high. The variability in environmental Hg may explain the high intra specific variability and occasional highly contaminated turtle seen in this and previous studies in the eastern Mediterranean Sea.

### Arsenic

The average levels of As found in our study are much lower than those, from Mediterranean Sea, reported in specimens from the east coast of Italy by Storelli et al.^36^ and^4^, and also from the east coast of Spain by Andreani et al.^22^ with regards to liver, kidney and muscle tissue. We observed the same pattern of distribution among the tissues as the latter (blood > muscle > liver > kidney > fat) (Fig. 2). In this pattern, the relatively high concentration of As in muscle tissue may indicate a specific metabolic mechanism of this element. The fluctuation in the results suggests differences between the base levels of As contamination in the different areas studied or in the feeding habits of the specimens^34^.

Some marine animals, such as molluscs and crustaceans, which are a part of the loggerhead sea turtle’s diet, may retain concentrations of this element^37^. In the case of As, one must take into account that only a small percentage of this metal (2–10%) is present in inorganic form and thus potentially toxic for organisms^38^.

The differences in concentration profiles in various tissues indicate sources of contamination related to diet and geographical area. In this context, the Mediterranean Sea can be considered a contamination hotspot as it is a semi-closed basin surrounded by 22 countries whose human activities and heavy maritime traffic lead to high pollution.

### Correlation between elements in the different tissues and organs

The correlation between the elements in the different tissues is shown in Table 3. In blood, arsenic was positively correlated with Pb (p < 0.001). In muscle tissue a positive correlation between As and Pb (p < 0.001) and between Cd and As (p < 0.05) and a negative correlation between Hg and both Cd and As (p < 0.05) were found to exist.

**Table 3:** Spearman correlation between heavy metals within the tissues of the loggerheads turtles (*Caretta* *caretta*) from the Egyptian coast.

Elements	Muscles	Liver	kidney	Fat	Blood
Cd	+ As–Hg	+ Pb + Hg	+ Pb	** + Pb**	** + As**
Pb	** + As**		+ Cd	+ Pb	
As	**− **Hg				
Hg		** + As**	**− As**	** + As**	

Bold: *p* < 0.001, unbold: *p* < 0.05.

The concentrations of Cd and both Pb and As were correlated in kidney tissue (p < 0.05), while a negative correlation between Hg and As (p < 0.001) was observed. In fat tissue a positive correlation between Cd and Pb and between Hg and As (p < 0.001) and between As and Pb (p < 0.05) was detected. This last correlation was also observed in liver tissue between As and Hg (p < 0.001). Finally, a statistically significant negative correlation between Pb in liver and kidney (p < 0.001), and also in fat and muscles, a statistically significant positive correlation between Cd in muscle and liver tissue (p < 0.001) exist.

Chronic exposure should give evidence of a parallel accumulation of As and both of Cd and Pb in tissues (Table 3), although we can observe a negative correlation between Hg and As in kidney and muscular tissue. This may be due to the lack of older specimens, whose tissue concentrations of As and Hg would allow us to observe a positive correlation between these two elements due to chronic exposure. On the other hand, the positive correlation between all elements in liver and fat may be indicative of chronic exposure.

### Potential health risk by turtle consumption

Sea turtle tissues are commonly used as food items in many communities worldwide, despite national regulations prohibiting such consumption. Although regulations prohibit the capture and consumption of sea turtles in Egypt, there are many people in coastal cities accept the consumption of sea turtle meat and also drink their blood. Blood-drinking was carried out mainly by women, mostly in the belief that it increases fertility. Thus, it is interesting and necessary to evaluate the quality of turtle’s meat as food. Levels of Pb found in muscle tissues from this study are above the established food safety guidelines from several nations (0.3–2.0 µg/g wet weight)^39–41^. Besides, the results reveal that Cd and Hg has the same manner of Pb i.e. they are above the established food safety guidelines from several nations (0.05 and 0.5 µg/g wet weight respectively). Consequently, from these results it is difficult to anticipate a health risk to indigenous communities that consume turtle meat. On the other hand, the serious damage comes from the continued drinking of sea turtles blood of some peoples, where the highest concentrations of toxic metals (in the present study) were concentrated in the turtle’s blood.

Alexandria, Egypt's fish market is one area where sea turtles are killed for meat or international trade. However, sea turtle conservationists and the Egyptian government are trying to monitor and secure sea turtle populations over the entire coast of Egypt. This will not be done only in the case of building a partnership with the fishermen, so is of crucial importance to provide a guideline for the planning process, decentralize the decision-making process, provide better communication with the fishermen, and enhance collaborative relationships with other local actors. At the same time helping to increase awareness, and motivating them to contribute in innovative ways that are characteristically of a social and economic nature, all of this may lead to quit with this habit.

## Data Availability

The datasets generated during and/or analyzed during the current study are not publicly available due to [after acceptance by the journal, it can be publicly available only] but are available from the corresponding author on reasonable request.
